# Adrenomedullin promotes the growth of pancreatic ductal adenocarcinoma through recruitment of myelomonocytic cells

**DOI:** 10.18632/oncotarget.10393

**Published:** 2016-07-04

**Authors:** Min Xu, Feifei Qi, Shaosen Zhang, Xuhui Ma, Shan Wang, Chunying Wang, Yan Fu, Yongzhang Luo

**Affiliations:** ^1^ The National Engineering Laboratory for Anti-Tumor Protein Therapeutics, School of Life Sciences, Tsinghua University, Beijing, China; ^2^ Beijing Key Laboratory for Protein Therapeutics, School of Life Sciences, Tsinghua University, Beijing, China; ^3^ Cancer Biology Laboratory, School of Life Sciences, Tsinghua University, Beijing, China

**Keywords:** pancreatic ductal adenocarcinoma, adrenomedullin, myelomonocytic cells, cell recruitment, tumor angiogenesis

## Abstract

Stromal infiltration of myelomonocytic cells is a hallmark of pancreatic ductal adenocarcinoma (PDAC) and is related to a poor prognosis. However, the detailed mechanism for the recruitment of myelomonocytic cells to pancreatic cancer tissue remains unclear. In the present study, pancreatic cancer cells secreted high levels of adrenomedullin (ADM), and CD11b^+^ myelomonocytic cells expressed all components of ADM receptors, including GPR182, CRLR, RAMP2 and RAMP3. ADM enhanced the migration and invasion of myelomonocytic cells through activation of the MAPK, PI3K/Akt and eNOS signaling pathways, as well as the expression and activity of MMP-2. ADM also promoted the adhesion and trans-endothelial migration of myelomonocytic cells by increasing expression of VCAM-1 and ICAM-1 in endothelial cells. In addition, ADM induced macrophages and myeloid-derived suppressor cells (MDSCs) to express pro-tumor phenotypes. ADM knockdown in tumor-bearing mice or administration of AMA, an ADM antagonist, significantly inhibited the recruitment of myelomonocytic cells and tumor angiogenesis. Moreover, *in vivo* depletion of myelomonocytic cells using clodronate liposomes suppressed the progression of PDAC. These results reveal a novel function of ADM in PDAC, and suggest ADM is a promising target in the treatment of PDAC.

## INTRODUCTION

Pancreatic ductal adenocarcinoma (PDAC) is a highly lethal malignancy associated with desmoplasia and a marked infiltration of myeloid cells into the tumor stroma [1]. In the United States, PDAC is estimated to have caused more than 40,000 deaths in 2015, which makes it the fourth leading cause of cancer-related death [2].

Tumor-associated myelopoiesis often causes abnormal production of immature myelomonocytic cells [3, 4]. The abundance of myelomonocytic cells, mainly monocyte-macrophage lineage, in tumor tissues correlates with inflammation and poor diagnosis [5, 6]. Tumor-secreted soluble factors, such as VEGF and GM-CSF, can stimulate these cells to differentiate into macrophages or result in the accumulation of myeloid-derived suppressor cells (MDSCs) [7, 8]. MDSCs reportedly promote tumor angiogenesis and growth through secretion of various cytokines, including VEGF and placental growth factor (PlGF), among others [9].

Adrenomedullin (ADM) is a 52-amino acid peptide initially isolated by Kitamura *et al.* from human pheochromocytoma [10]. First identified as a potent vasodilator belonging to the calcitonin superfamily, ADM is now known to be a multifunctional peptide involved in angiogenesis, cell proliferation, and anti-inflammation, acting mainly through binding to its receptor complexes composed of calcitonin receptor-like receptor (CRLR) and specific receptor activity modifying proteins RAMP2 and RAMP3 [11–13]. G protein-coupled receptor 182 (GPR182) is thought to be another ADM receptor that mediates cell proliferation and invasion [14]. When ADM binds to its receptors in human umbilical vein endothelial cells (HUVECs), activation of adenylate cyclase and protein kinase A (PKA) leads to cAMP production and, in turn, increases expression of VCAM-1, ICAM-1 and E-selectin [15, 16]. ADM also contributes to vascular regeneration or angiogenesis through activation of PI3K/Akt, MAPK and endothelial nitric oxide synthase (eNOS) signaling pathways [17–19]. Furthermore, ADM is overexpressed in various types of cancer, including pancreatic and prostate cancer, and appears to act as an autocrine and/or paracrine mediator that promotes tumor growth [14, 20–22]. Blocking ADM secretion from cancer cells or tumor-associated macrophages (TAMs) using a specific antibody or ADM antagonist (AMA) inhibits tumor angiogenesis and growth [19, 21, 23].

During tumor development, large numbers of myeloid cells infiltrate tumors. In the center of tumors, there is often a hypoxic microenvironment that can upregulate the expression of ADM [24]. Among the infiltrating myeloid cells, most of them are TAMs which have been skewed towards a pro-tumor M2 phenotype. And they preferentially localize within the hypoxic areas of tumors [25]. We therefore tested whether ADM can recruit myelomonocytic cells to tumors and influence the phenotype of myeloid cells to promote tumor angiogenesis and growth.

Here, we demonstrate that the level of ADM expression negatively correlates with disease-free survival in pancreatic cancer patients. And there is a positive correlation between ADM expression levels and the density of myelomonocytic cells. ADM promotes the migration and invasion of myelomonocytic cells through activation of MAPK, PI3K/Akt and eNOS signaling pathways. It also promotes myelomonocytic cell-endothelial cell adhesion and subsequent trans-endothelial migration. Furthermore, ADM induces macrophages and MDSCs to express pro-tumor phenotypes, finally contributing to tumor angiogenesis and growth. Collectively, these results provide another insight for how ADM contributes to pancreatic cancer growth and unravelling a promising way for pancreatic cancer treatment.

## RESULTS

### ADM is highly expressed in pancreatic cancer tissues and its level correlates with the abundance of CD11b^+^ myelomonocytic cells

**Figure 1:**
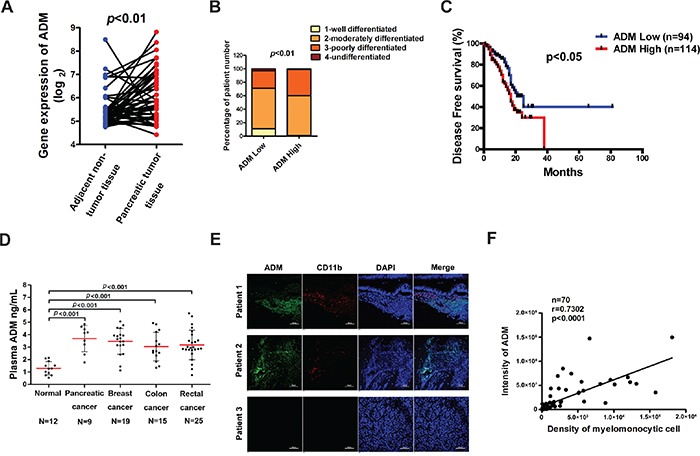
ADM is highly expressed in pancreatic cancer tissues and its level correlates with the abundance of CD11b+ myelomonocytic cells **A.** Comparison of mRNA levels of ADM between pancreatic cancer tissues and adjacent non-cancer tissues (n=42); *p* value, ANOVA. **B.** Correlation between mRNA levels of ADM and pancreatic cancer differentiation (n=200); *p* value, Chi-square test. **C.** Kaplan-Meier survival analysis of pancreatic cancer patients classified by ADM expression (n=94 for ADM low group, n=114 for ADM high group); *p* value, log-rank (Mantel-Cox) test. **D.** ADM levels in the plasma of normal people and cancer patients were detected by ELISA; *p* values, Student's *t* test. **E.** Representative images of immunofluorescence of ADM and CD11b in human pancreatic cancer tissues (n=70); Scale bar=100 μm. **F.** Quantified results showing the correlation between ADM expression levels and the density of CD11b^+^ myelomonocytic cells in (E) by Pearson's correlation coefficient (*r* =0.7302).

ADM has been previously reported to be overexpressed in several types of cancer, such as colorectal cancer, pancreatic cancer, clear cell renal cell carcinoma (RCC) and so on [14, 26, 27]. To further confirm the clinical significance of ADM expression, we analyzed the pancreatic cancer data set and discovered that mRNA levels of ADM were significantly higher in pancreatic cancer tissues than those in adjacent normal tissues (Figure 1A). We also correlated ADM levels with clinicopathological status of pancreatic cancer patients and found that mRNA levels of ADM did not correlate with gender, age, or stage of lymph node metastasis (Supplementary Table S1). But patients with low ADM expression exhibited better tumor differentiation than those with high ADM levels (Figure 1B, Supplementary Table S2). Strikingly, Kaplan-Meier survival curve demonstrated that pancreatic cancer patients with high ADM levels had poor disease free survival (Figure 1C), indicating that ADM was a prognostic factor for pancreatic cancer. Additionally, the protein levels of ADM in the plasma of patients with different types of cancer, pancreatic cancer included, were significantly higher than those in healthy people (Figure 1D, Supplementary Table S3). Our results also revealed that plasma ADM levels positively correlated with the malignancy in breast cancer and colorectal cancer (Supplementary Figure S1A). The ROC curves uncovered that ADM in plasma had a good sensitivity and specificity to distinguish cancer patients from healthy people (Supplementary Figure S1B).

A marked infiltration of myeloid cells was observed in pancreatic cancer [1]. To detect the correlation between ADM and myelomonocytic cells, we stained the human pancreatic cancer tissue array with ADM and myelomonocytic cell marker CD11b antibodies. The expression levels of ADM in pancreatic cancer tissues positively correlated with the density of CD11b^+^ cells (*r*=0.7302) (Figure 1E, 1F). Taken together, these results demonstrate that ADM negatively correlates with prognosis of pancreatic cancer and positively correlates with the abundance of myelomonocytic cells in human pancreatic cancer tissues.

### ADM promotes the migration and invasion of myelomonocytic cells

**Figure 2:**
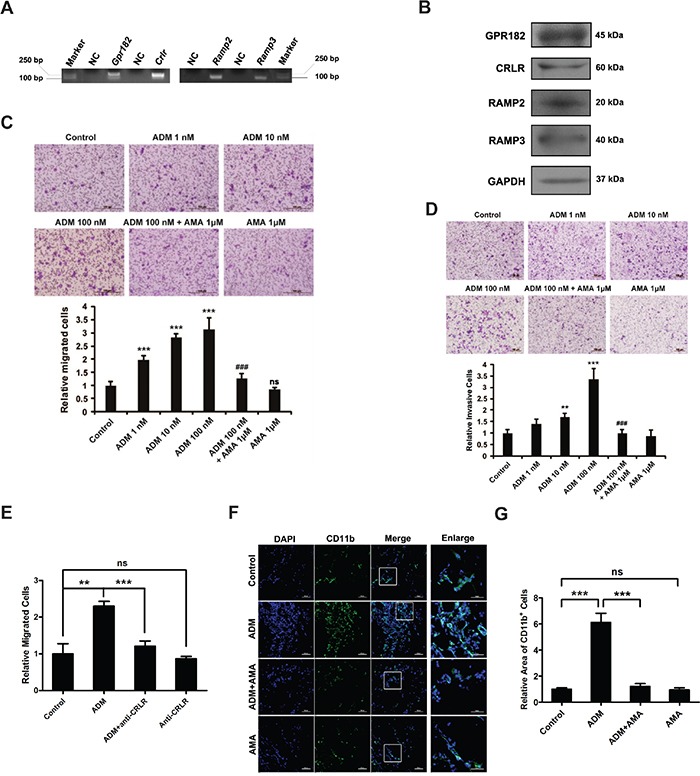
ADM promotes the migration and invasion of myelomonocytic cells **A.** RT-PCR showing the expression of ADM receptor components in CD11b^+^ myelomonocytic cells. **B.** Western blot showing the expression of ADM receptor components in CD11b^+^ myelomonocytic cells. **C.** Representative images and quantitation of ADM-induced myelomonocytic cell migration determined by modified Boyden chamber assay; Scale bar=100 μm. **D.** Representative images and quantitation of ADM-induced myelomonocytic cell invasion; Scale bar=100 μm. **E.** Quantitation of the effect of CRLR blockade with its antibody on ADM-induced myelomonocytic cell migration. **F.** Representative images of the density of CD11b^+^ myelomonocytic cells in Matrigel plugs; Scale bar=100 μm. **G.** Quantitation of myelomonocytic cells density in Matrigel plugs. Five independent fields were counted in each section. Data were representative of means ± SD from at least three independent experiments. *p* value: Student's *t*-test; ^**^p < 0.01, ^***^p < 0.001, ^###^*p* < 0.001, ns: not significant.

To find out the possible interaction between ADM and myelomonocytic cells, we firstly detected different ADM receptor components in myelomonocytic cells and found that GPR182, CRLR, RAMP2 and RAMP3 were all expressed in these cells (Figure 2A, 2B and S2A). Migration and invasion assays were then applied to examine the effect of ADM on the recruitment of myelomonocytic cells. Upon addition of ADM to the lower chamber, cell migration and invasion were significantly increased in a dose-dependent manner. However, these effects could be blocked by AMA (Figure 2C, 2D). To find out the association between myelomonocytic cells recruitment and ADM receptor, we used CRLR antibody to block the ADM receptor in myelomonocytic cells and Raw 264.7 cells and found that ADM-induced migration could be reversed (Figure 2E, S2B-S2D). Stained sections from Matrigel plugs also revealed that ADM increased the recruitment of CD11b^+^ myelomonocytic cells and AMA significantly blocked this effect (Figure 2F, 2G). Collectively, these results suggest that ADM promotes the recruitment of myelomonocytic cells by enhancing their migration and invasion abilities.

### ADM activates intracellular signaling pathways and increases the expression and activity of MMP-2 in myelomonocytic cells

**Figure 3:**
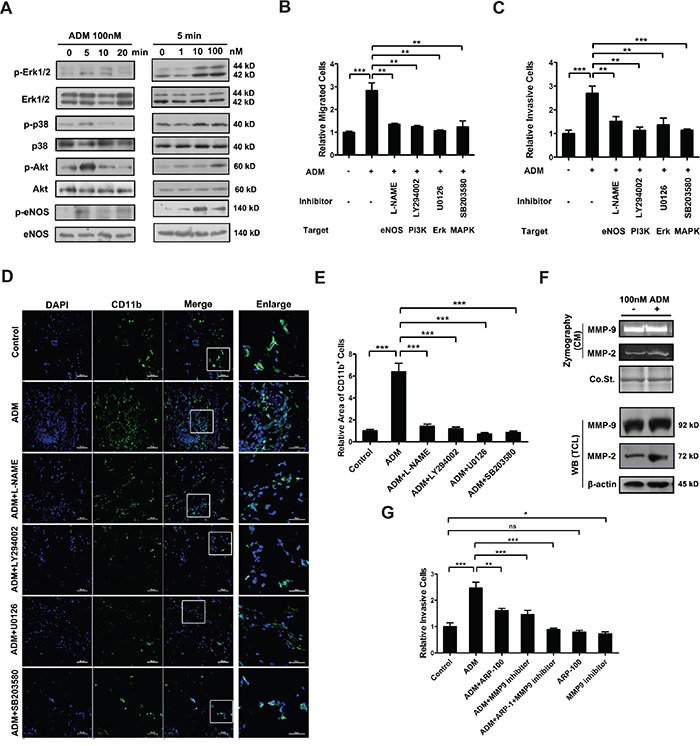
ADM activates intracellular signaling pathways and increases the expression and activity of MMP-2 in myelomonocytic cells **A.** Western blot showing the effect of ADM on the activation of p38, Erk1/2, Akt, and eNOS. Isolated CD11b^+^ myelomonocytic cells were cultured in medium containing 1% FBS for 12 h and then treated with 100 nM ADM for indicated time or treated with different concentrations of ADM for 5 min. **B.** The migration ability of myelomonocytic cells in transwell assay upon pharmacological inhibition of eNOS, PI3K, Erk1/2, and MAPK pathways in the presence of ADM. Myelomonocytic cells were pre-treated with 100 nM ADM and subsequently treated with 10 μM eNOS inhibitor L-NAME or 10 μM PI3K inhibitor LY294002 or 10 μM Erk1/2 inhibitor U0126 or 10 μM MAPK inhibitor SB203580. **C.** The invasion ability of myelomonocytic cells in transwell assay upon pharmacological inhibition of eNOS, PI3K, Erk1/2, and MAPK pathways in the presence of ADM. **D.** Representative images of CD11b^+^ myelomonocytic cells recruited to Matrigel plugs containing 10 μM L-NAME or 10 μM LY294002 or 10 μM U0126 or 10 μM SB203580 in the presence of ADM; Scale bar=100 μm and 50μm for enlarged field. **E.** Quantitation of the density of myelomonocytic cells in (D). Five independent fields were counted in each section. **F.** Gelatin zymography assay (upper) and Western blot (lower) showing the effect of ADM on the expression and activity of MMP-2 and MMP-9 in myelomonocytic cells. **G.** The invasion ability of myelomonocytic cells in transwell assay upon pharmacological inhibition of MMP-2 and MMP-9 in the presence of ADM. Myelomonocytic cells were treated with 100 nM ADM either alone or in the presence of 25 μM MMP-2 inhibitor ARP-100 or 5μM MMP-9 inhibitor or both inhibitors. Data were representative of means ± SD from at least three independent experiments. *p* value: Student's *t*-test; ^**^p < 0.01, ^***^p < 0.001, ns: not significant.

Increased cell motility is often caused by activated intracellular signaling pathways. As expected, Western blot showed that ADM treatment induced phosphorylation of p38, Erk1/2, Akt and eNOS (Figure 3A). To evaluate the contributions of PI3K/Akt, MAPK and eNOS signaling pathways to ADM-induced chemotaxis, inhibitors of PI3K (LY294002), p38 (SB203580), Erk1/2 (U0126) and eNOS (L-NAME) were used to treat myelomonocytic cells, respectively. After the treatment, ADM-induced migration and invasion were both significantly attenuated (Figure 3B, 3C and S3A-S3D). And these inhibition effects were not attributed to cell apoptosis induced by these inhibitors (Supplementary Figure S3E, S3F). These inhibitors could also significantly inhibit ADM-induced recruitment of myelomonocytic cells *in vivo* (Figure 3D, 3E). Furthermore, both gelatin zymography assay and Western blot results showed that ADM upregulated the expression and activity of MMP-2 but not MMP-9 in myelomonocytic cells (Figure 3F). ARP-100 is a well-known selective inhibitor of MMP-2 [28, 29]. After the administration of ARP-100 to myelomonocytic cells treated by ADM, cell invasion triggered by ADM was inhibited. If we blocked the activities of MMP-2 and MMP-9 together, the inhibition effect was more significant (Figure 3G). Taken together, these results suggest that activated MAPK, PI3K/Akt and eNOS signaling pathways and increased MMP-2 contribute to the ADM-enhanced migration and invasion of myelomonocytic cells.

### ADM enhances the adhesion of myelomonocytic cells to endothelial cells and extracellular matrix (ECM)

**Figure 4:**
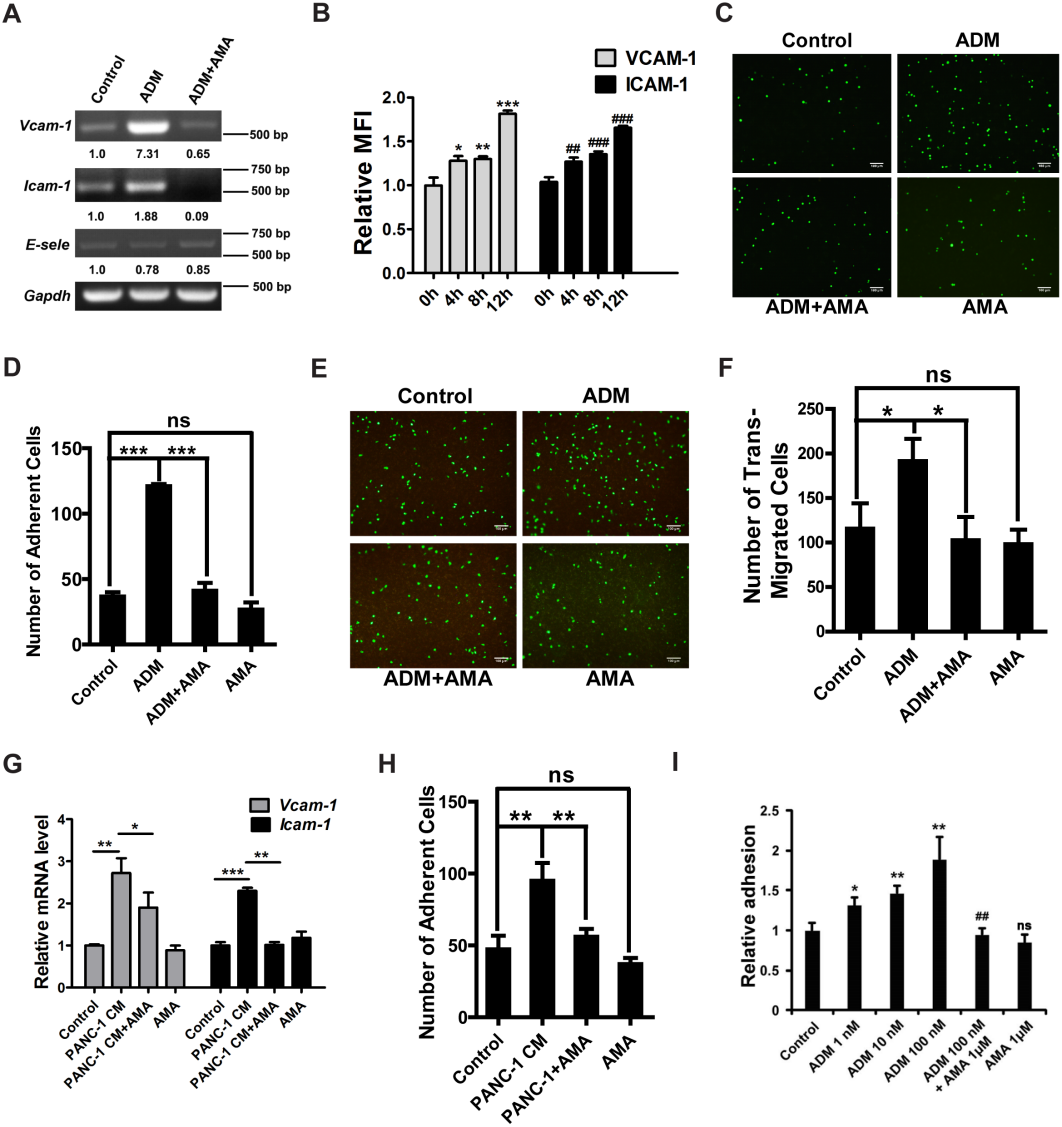
ADM enhances the adhesion of myelomonocytic cells to endothelial cells and extracellular matrix (ECM) **A.** RT-PCR results showing the effect of ADM on the expression of VCAM-1, ICMA-1 and E-selectin in MS1. MS1 was starved in serum-free medium for 24 h and then treated with 100 nM ADM for 4 h. In AMA treatment group, MS1 was pre-treated with 1 μM AMA for 1 h and then treated with ADM for 4 h. GAPDH was used as a loading control. **B.** Relative MFI of VCAM-1 and ICAM-1 in MS1 treated with ADM for 4, 8, and 12 h. **C.** Representative images of ADM inducing myelomonocytic cells to adhere to MS1 either alone or in the presence of AMA; Scale bar=100 μm. **D.** Quantified results of (C). **E.** Representative images of trans-endothelial migration of myelomonocytic cells; Scale bar=100 μm. **F.** Quantified results of (E). **G.** qRT-PCR results showing the effect of PANC-1 CM on the expression of VCAM-1 and ICAM-1 in MS1. GAPDH was used as the control. **H.** Quantified results of myelomonocytic cell-MS1 adhesion upon treatment with PANC-1 CM alone or in the presence of AMA. **I.** Quantitation of myelomonocytic cells adhering to ECM upon the treatment of ADM. Data were representative of means ± SD from at least three independent experiments. *p* value: Student's *t*-test; ^*^p < 0.05, ^**^p < 0.01, ^***^p < 0.001, ^##^*p* < 0.01, ^###^*p* < 0.001, ns: not significant.

VCAM-1, ICAM-1 and E-selectin are key adhesion molecules which mediate the interaction between myeloid cells and endothelial cells. To investigate the effects of ADM on adhesion process, we used ADM to treat mouse pancreas islet endothelial cell MS1 which expressed all ADM receptor components (Supplementary Figure S4A-S4C). RT-PCR and flow cytometry analyses showed that ADM upregulated expression of VCAM-1 and ICAM-1 but not E-selectin in MS1 (Figure 4A, 4B and S4D). There was a 3-fold increase in the adhesion of GFP^+^ myelomonocytic cells to ADM-treated MS1 (Figure 4C, 4D). Similar results were also obtained in HUVECs. After treating HUVECs with ADM, VCAM-1 and ICAM-1 were upregulated and more human monocyte U937 cells adhered to HUVECs (Supplementary Figure S4E-S4G). After adhering to endothelial cells, more myelomonocytic cells extravasated through endothelial cells in ADM-treated group but AMA inhibited this process (Figure 4E, 4F).

To further confirm these results, we treated MS1 with the conditioned medium (CM) of PANC-1 cells which expressed the highest level of ADM among different PDAC cell lines (Supplementary Figure S4H, S4I), and found that VCAM-1 and ICAM-1 were upregulated but AMA reversed this effect (Figure 4G). More myelomonocytic cells attached to the PANC-1 CM-treated MS1 while pre-treatment with AMA blocked this effect (Figure 4H, S4J). Also upregulated in myelomonocytic cells was integrin α5 (Supplementary Figure S4K) but not integrin α2 (data not shown). Increased integrin α5 assisted myelomonocytic cells in adhering to ECM. After ADM treatment, the number of myelomonocytic cells adhering to ECM was 1.8-fold increase (Figure 4I). Downstream signaling molecules of integrin α5 such as FAK and Src were both activated after ADM treatment in a time-dependent manner (Supplementary Figure S4L).

Collectively, these data illustrate that ADM promotes the recruitment of myelomonocytic cells through enhancing the cell-cell adhesion, trans-endothelial migration and cell-ECM adhesion.

### Myelomonocytic cells recruited by ADM promote tumor angiogenesis and growth

**Figure 5:**
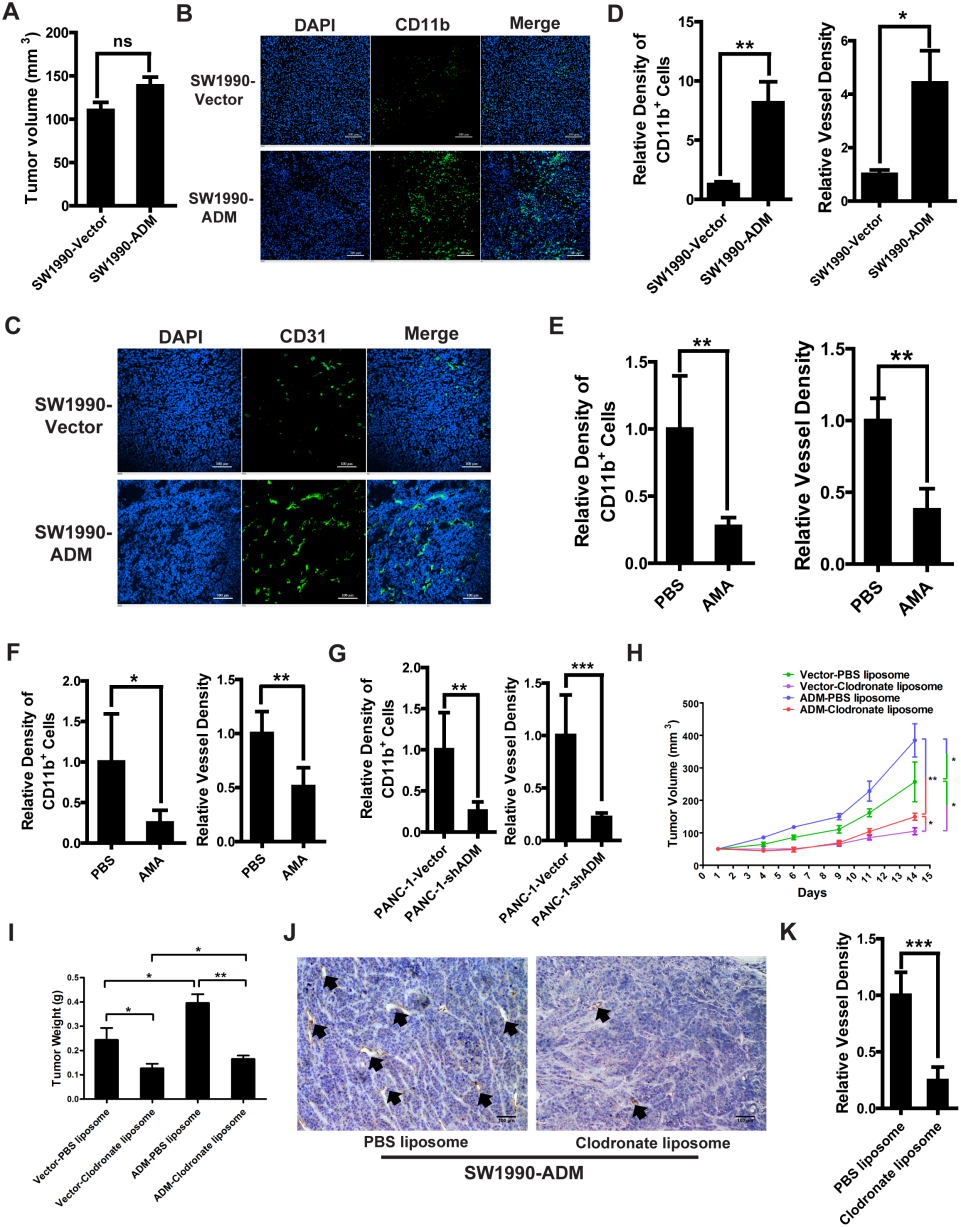
Myelomonocytic cells recruited by ADM promote tumor angiogenesis and growth **A.** Tumor volumes of SW1990-Vector and SW1990-ADM after 1 week (n=6 mice/group). **B.** Representative images of immunofluorescence showing the density of CD11b^+^ myelomonocytic cells in SW1990 tumor tissues; Blue: DAPI, green: CD11b; Scale bar=100 μm. **C.** Representative images of immunofluorescence showing the tumor angiogenesis in SW1990 tumor tissues through detecting CD31^+^ vessels; Blue: DAPI, green: CD31; Scale bar=100 μm. **D.** Quantified results of myelomonocytic cells recruitment (left) and tumor angiogenesis (right). **E.** Quantitation of the effect of AMA on myelomonocytic cells recruitment (left) and tumor angiogenesis (right) in SW1990-ADM tumor-bearing mice. 7 days after tumor implantation, mice were divided into two groups randomly and treated with PBS or AMA (6 μg/day). On day 12, mice were sacrificed and CD11b and CD31 were stained in cryo-sections. **F.** Quantitation of the effect of AMA on myelomonocytic cells recruitment (left) and tumor angiogenesis (right) in PANC-1 tumor-bearing mice. **G.** Quantitation of the effect of ADM knockdown on myelomonocytic cells recruitment (left) and tumor angiogenesis (right) in PANC-1 tumor-bearing mice. **H.** SW1990-Vector and SW1990-ADM tumor growth in mice treated with PBS or clodronate liposomes. 100μL PBS or clodronate liposomes were injected into the mice i.v. 6 days before the tumor implantation (n=6 mice/group), followed by treatment every 3 days. On day 14, mice were sacrificed and tumors were weighted. **I.** SW1990-Vector and SW1990-ADM tumor weights in mice treated with PBS or clodronate liposomes. **J.** IHC analysis of CD31^+^ vessels in SW1990-ADM tumor tissues treated with PBS or clodronate liposomes; Scale bar=100 μm. **K.** Quantified result of vessel density in (J). Five independent fields were observed in each section. Data were representative of means ± SD or SEM for animal experiment. *p* value: Student's *t*-test; ^*^p < 0.05, ^**^p < 0.01, ^***^p < 0.001, ns: not significant.

As human pancreatic adenocarcinoma cell SW1990 had the lowest expression of ADM among different PDAC cell lines (Supplementary Figure S4H, S4I), we constructed the SW1990-ADM cell line which stably overexpressed ADM (Supplementary Figure S5A). To investigate whether ADM could increase the recruitment of myelomonocytic cells *in vivo*, we implanted SW1990-Vector and SW1990-ADM cells subcutaneously into mice and evaluated the density of CD11b^+^ myelomonocytic cells in tumor tissues. One week after implantation, tumor volumes in both groups displayed no significant differences so the impact of tumor volume difference could be excluded (Figure 5A). More CD11b^+^ myelomonocytic cell were recruited to SW1990-ADM tumors compared to SW1990-Vector tumors, accompanied by increased tumor angiogenesis (Figure 5B-5D). We also observed that these myelomonocytic cells located closely to blood vessels and more myelomonocytic cells attached to endothelial cells in SW1990-ADM group (Supplementary Figure S5B). To further confirm the effect of ADM on cell recruitment, PBS or AMA (6 μg/day) was intraperitoneally injected into SW1990-ADM tumor-bearing mice for 6 consecutive days. We found that the tumor growth was inhibited in the AMA-treated group (Supplementary Figure S5C). The recruitment of CD11b^+^ myelomonocytic cells and tumor angiogenesis were also decreased in AMA-treated group compared to those in PBS-treated group (Figure 5E, S5D and S5E).

Moreover, similar results were obtained in PANC-1 tumor-bearing mice. After treating these mice with AMA, tumor growth was inhibited (Supplementary Figure S6A). The recruitment of CD11b^+^ cells and tumor angiogenesis were also significantly decreased (Figure 5F, S6B and S6C). The lentivirus system was used to stably knock down ADM in PANC-1 (PANC-1-shADM) (Supplementary Figure S6D). Then we implanted PANC-1-Vector and PANC-1-shADM cells into mice subcutaneously. After 1 week, no significant differences of tumor volumes were seen between these two groups (Supplementary Figure S6E). But the density of CD11b^+^ cells and tumor angiogenesis were obviously decreased in the PANC-1-shADM group compared to those in PANC-1-Vector group (Figure 5G, S6F and S6G).

To evaluate whether the inhibition of tumor angiogenesis and growth were partly mediated by the decreased recruitment of myelomonocytic cells, clodronate liposomes were administered to deplete CD11b^+^ myelomonocytic cells in SW1990-Vector and SW1990-ADM tumor-bearing mice. The depletion efficiency was almost 95% after treating with clodronate liposomes. Among the CD11b^+^ myelomonocytic cells, F4/80^+^ macrophages were decreased most significantly (Supplementary Figure S5F, S5G). Within SW1990-ADM groups, tumor growth and angiogenesis were both inhibited significantly after clodronate liposome treatment. Moreover, we found that myelomonocytic cells depletion displayed a stronger effect on tumor angiogenesis and growth compared to the pro-angiogenic effect of ADM (Figure 5H-5K). Clodronate liposome treatment also significantly inhibited tumor growth and angiogenesis in PANC-1 tumor-bearing mice (Supplementary Figure S6H-S6J). MTT assay showed that clodronate liposomes wouldn't directly affect the cell viability of SW1990-ADM and PANC-1 (Supplementary Figure S5H, S6K).

In summary, these findings demonstrate that ADM can promote the growth of pancreatic cancer in a new way through directly recruiting myelomonocytic cells to the tumor.

### ADM induces macrophages and myeloid-derived suppressor cells to express pro-tumor phenotypes

**Figure 6:**
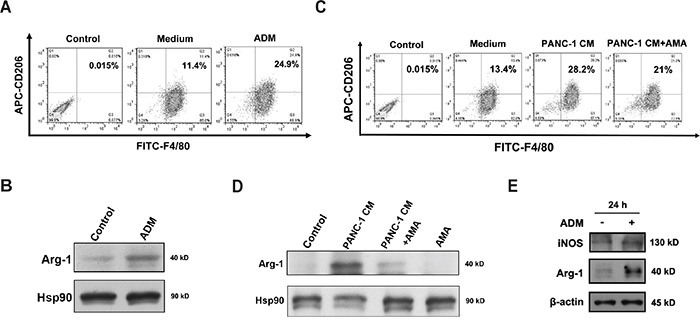
ADM induces macrophages and myeloid-derived suppressor cells to express pro-tumor phenotypes **A.** Flow cytometry analysis of the polarization of BMDMs treated with 100 nM ADM for 24 h. Numbers indicated the percentage of F4/80^+^CD206^+^ cells among the total BMDMs. **B.** Western blot showing the effect of ADM on Arg-1 expression in BMDMs. **C.** Flow cytometry analysis of the polarization of BMDMs treated with PANC-1 CM alone or in the presence of AMA for 24 h. Numbers indicated the percentage of F4/80^+^CD206^+^ cells among the total BMDMs. **D.** Western blot showing the effect of PANC-1 CM on Arg-1 expression in BMDMs. **E.** Western blot indicating protein levels of Arg-1 and iNOS in MDSCs treated with ADM for 24 h.

As infiltrating myelomonocytic cells are mainly monocyte-macrophage lineage, most of them differentiate into macrophages or accumulate in the form of MDSCs after entrance into the tumor microenvironment. Then we detected whether ADM could influence the phenotypes of macrophages and MDSCs. After treating bone marrow derived-macrophages (BMDMs) with ADM for 24 h, M2-specific surface marker CD206 was detected by flow cytometry. The percentage of F4/80^+^CD206^+^ cells was increased by about 2-fold and Western blot also showed Arg-1, a M2 marker, was upregulated (Figure 6A, 6B). To investigate whether pancreatic cancer could alter the polarization of macrophage depending on ADM, we incubated BMDMs with PANC-1 CM and found that the percentage of F4/80^+^CD206^+^ cells was increased while AMA partially blocked this effect (Figure 6C). Western blot result also uncovered that Arg-1 was significantly increased after the treatment of PANC-1 CM but AMA reversed this effect (Figure 6D). As we all know, TAMs which are skewed towards the pro-tumor M2 type preferentially localize within hypoxic areas of tumor. To verify the effect of hypoxia on the process of ADM educating macrophages towards pro-tumor phenotype, we treated BMDMs with ADM combined with the hypoxia-mimetic agent cobalt chloride (CoCl_2_). CoCl_2_ upregulated the expression of HIF-1α and ADM in BMDMs (Supplementary Figure S7A, S7B). However, it didn't show any effects on the process of ADM stimulating macrophages to pro-tumor phenotypes (Supplementary Figure S7C-S7E). We also treated CD11b^+^Gr1^+^ MDSCs with ADM and protein levels of Arg-1 and iNOS were both upregulated (Figure 6E). To summarize, our results prove that ADM secreted by pancreatic cancer cells can transform the phenotypes of macrophages and MDSCs towards pro-tumor types.

## DISCUSSION

ADM is highly expressed in PDAC, and large numbers of myelomonocytic cells infiltrate the stromal compartment of PDAC. In the present study, we observed that the level of ADM expression correlates positively with the density of myelomonocytic cells in human pancreatic cancer tissues. In addition to enhancing the motility of myelomonocytic cells, ADM promoted the adhesion of myelomonocytic cells to endothelial cells and ECM and induced myeloid cells to express pro-tumor phenotypes. These recruited myelomonocytic cells then contributed to angiogenesis and growth of PDAC.

Previous reports showed that a high density of bone marrow-derived cells is associated with poor disease-free survival in different cancer types [5, 6]. Our findings indicate that high ADM expression is associated with poor disease-free survival, and that there is a positive correlation between ADM levels and the density of CD11b^+^ myelomonocytic cells in human PDAC tissues. So the abundance of myelomonocytic cells may be also an indicator for disease-free survival in PDAC. Myelomonocytic cells, mainly monocyte-macrophage lineage, express all ADM receptor components. Notably, ADM receptor signaling enhance endothelial cell migration by activating downstream MAPK, PI3K/Akt and eNOS signaling [18, 19]. These signaling pathways also mediated ADM-induced recruitment of myelomonocytic cells *in vitro* and *in vivo*. Besides, MMP-2 but not MMP-9 upregulated by ADM will facilitate the degradation of ECM and further contributes to myelomonocytic cell invasion.

Leukocyte-endothelial interaction is the prerequisite step for leukocyte infiltration of inflamed tissues. Myelomonocytic cells use the same process to enter the tumor microenvironment. Normally, these interactions are mediated by sialyl Lewis X or integrins on leukocytes and adhesion molecules such as VCAM-1 and ICAM-1 on endothelial cells [30, 31]. ADM reportedly upregulates expression of VCAM-1 and ICAM-1 in HUVECs [15]. VCAM-1 and ICAM-1 are known to mediate firm adhesion through binding integrin α1β7, LFA-1 and Mac-1 [30, 32]. We observed that in MS1, ADM increased expression of VCAM-1 and ICAM-1, which in turn mediate firm cell-cell adhesion to promote trans-endothelial migration. Though it's difficult to detect the adhesion of myelomonocytic cells to endothelial cells inside the intact blood vessel lumen, we observed that most myelomonocytic cells lay closely to blood vessels in the tumor tissues and more myelomonocytic cells adhered to endothelial cells in the SW1990-ADM group compared to that in SW1990-Vector group. Also upregulated in myelomonocytic cells was integrin α5. Downstream signaling of integrin α5 leads to activation of FAK and Src, which reportedly regulate adhesion disassembly to promote cell motility [33]. Thus activated FAK and Src may also contribute to myelomonocytic cell invasion.

Within the tumor microenvironment, most myelomonocytic cells exist as MDSCs or further differentiate into macrophages. MDSCs and TAMs are well-known to take part in immune suppression and angiogenesis in tumor-bearing mice [9, 34]. Our study revealed that treating MDSCs with ADM upregulated the expression of Arginase-1 and iNOS, which are linked to immunosuppression [35, 36]. So ADM may promote pancreatic cancer cell survival via assisting tumor cells in evading the immunological surveillance. Our laboratory previously demonstrated that ADM altered the polarization of Raw264.7 in an autocrine manner [19]. Here we observed that upon treating BMDMs with PANC-1 CM, the percentage of M2 type macrophages was increased and that AMA partially inhibited this process. It suggests there must be other factors interacting with ADM to regulate the activity and function of TAMs together.

Similar to other pro-angiogenic factors like VEGFA and PlGF secreted by tumor cells [37–40], ADM promotes pancreatic cancer growth through stimulating tumor angiogenesis and recruiting myelomonocytic cells. However, due to myeloid cells also having pro-angiogenic effects, it is difficult to say between neovascularization and myeloid cell recruitment which one has a greater impact on tumor growth. To test that idea, we attempted to dissociate neovascularization and myelomonocytic cells recruitment in the process of tumor growth. Before implanting SW1990-Vector and SW1990-ADM tumors, clodronate liposomes were injected, which blocked recruitment of myelomonocytic cells. Under that condition, we only focused on the neovascularization effect of ADM at early stage between SW1990-Vector and SW1990-ADM groups. Although recruitment of myelomonocytic cells was blocked, SW1990-ADM tumors still secreted ADM to enhance neovascularization and promote tumor growth. Within the SW1990-Vector groups (PBS and clodronate liposome), we only focused on the effect of myelomonocytic cell recruitment. Myelomonocytic cell depletion significantly inhibited SW1990-Vector tumor growth. And we observed that the impact of myelomonocytic cells depletion on tumor volume/weight was bigger than that of neovascularization triggered by ADM. Lin *et al.* once reported that reintroduced VEGFA into the mammary epithelium of macrophage depletion mice restores the delayed tumor progression [41]. In our study, the pro-angiogenic factor VEGFA was also used to stimulate neovascularization after myelomonocytic cells depletion. Compared to the neovascularization effect of VEGFA, myelomonocytic cell depletion by clodronate liposomes had a greater impact on tumor growth (data not shown). Previous studies showed that in addition of factors like VEGFA and bFGF, which promote tumor angiogenesis, myelomonocytic cells also produce EGF, HGF, MMP-9 and other cytokines that directly enhance the tumor cell survival, growth and invasion [6, 9]. Pancreatic cancer progression is the product of multiple actions within this very complex microenvironment and arises in part through the actions of myelomonocytic cells. So the driving force of tumor growth is a comprehensive result. Though at present we can't define that myelomonocytic cells are the most important driving force of tumor growth, it provides us a new insight that myelomonocytic cell recruitment is a novel function of ADM contributing to PDAC growth.

In summary, these findings provide a new insight into the roles of ADM in pancreatic cancer and how interruption of these processes may provide a novel and potentially effective way to treat pancreatic cancer.

## MATERIALS AND METHODS

For more detailed information, please see the supplementary materials and methods.

### Cells and reagents

PANC-1, SW1990, HEK293T, MS1, Raw 264.7 cells were obtained from the ATCC and maintained in DMEM supplemented with 10% FBS. Peptides were synthesized in Chinese Peptide Company (Zhejiang, China). Small molecule inhibitors such as L-NAME, LY294002, U0126 and SB203580 were from Sigma-Aldrich (St. Louis, MO). ARP-100 and MMP-9 inhibitor were from Santa Cruz Biotechnology (Santa Cruz, CA). Antibodies used in this study were listed in the supplementary materials and methods.

### Human tissue samples

Human pancreatic cancer tissue assay was purchased from Alenabio (Xi'an, China). Tissue microarray was co-immunostained with ADM and CD11b antibodies according to the protocol of immunofluorescence. Fluorescent images were captured by Nikon A1 laser scanning confocal microscope. ADM expression levels and the density of CD11b^+^ myelomonocytic cells were then quantified with Nikon image software NIS-Elements AR 3.0. Finally, Pearson's correlation coefficient was used to analyze the correlation between ADM expression levels and the density of CD11b^+^ myelomonocytic cells.

### Quantitation of ADM by ELISA

The concentration of ADM in plasma and conditioned medium was quantified using the ADM ELISA Kit (Ground Biotechnology Diagnosticate, China) according to the manufacturer's instructions.

### Cell sorting and analysis

Bone marrow was flushed from both femurs and tibias of C57BL/6 mice with 10 mL RPMI 1640. Then cells were treated with RBCs lysis buffer and centrifuged at 1,500 rpm for 3 min. The remaining cells were washed and passed through the 70 μm filter. Prepared cells were incubated with fluorescein-conjugated primary antibodies for 30 min at 4°C. Cells were washed with cold PBS twice and sorted or analyzed by FACSAria III (BD Biosciences, San Jose, CA).

### Immunohistochemistry and immunofluorescence

Immunohistochemistry was conducted with DAB kit (Zhongshan Golden Bridge, China) according to the manufacturer's instructions. Slides were observed under Olympus IX71 optical microscope.

Cells or cancer tissues were stained with indicated primary antibodies and then fluorescein-conjugated secondary antibodies as previously described [42]. Fluorescent images were captured by Nikon A1 laser scanning confocal microscope and analyzed with Nikon image software NIS-Elements AR 3.0.

### mRNA and protein analysis

Quantitative real-time PCR (qRT-PCR) was used to analyze the mRNA expression level. RT-PCR was conducted with standard PCR conditions and products were run on 1% agarose gel. All primers were listed in the Supplementary Table S4. Protein expression was analyzed by immunobloting.

### Cell functional assays

Transwell migration and invasion assays [42], cell-cell adhesion assay [43], cell-extracellular matrix (ECM) adhesion assay [44] and trans-endothelial migration assay [40] were performed as previously described. The number of migrated or adhered cells was measured by Image-Pro Plus 6.0 software (Media Cybernetics).

### Matrigel plug assay

500 μL Matrigel (Corning, NY, USA) containing ADM (500 ng/mL), AMA (1 μg/mL) or small molecule inhibitors was injected subcutaneously into the abdominal midline of BALB/c mice (Vital River, China). After 8 days, plugs were dissected and subjected to the immunofluorescent analysis. The density of CD11b^+^ cells was evaluated in 5 independent fields.

### Animal studies

All animal studies were approved by the Institutional Animal Care and Use Committees of Tsinghua University. Pancreatic cancer cells were inoculated subcutaneously into the right flank of 5 weeks old male BALB/c nude mice (Vital River, China). Then these mice were separated into 2 groups randomly (n=6 mice/group). For the AMA treatment group, mice were treated daily with intraperitoneal injection of 6 μg/day AMA or PBS for indicated days. For PANC-1-shADM and SW1990-ADM groups, tumors were allowed to grow for 1 week. Tumor growth was monitored every day. Tumor volumes were calculated by the formula: volume=0.52ab^2^ (“a” indicates the long diameter and “b” is the short diameter).

### Clodronate encapsulation

Clodronate liposomes were prepared as reported [45]. Clodronate was encapsulated in liposome which consisted of cholesterol (Sigma-Aldrich) and phosphatidylcholine (Lipoid, Germany) under the protection of argon.

### Human gene expression data sets

The analysis was conducted as previously reported [46]. Data of gene expression of pancreatic cancer patients were from International Cancer Genome Consortium (ICGC) pancreatic cancer project (Code: PACA-AU) and Gene Expression Omnibus database (GSE28735 and GSE50827) [47, 48]. For survival analysis, K-means clustering algorithm was used to cluster these gene expression data into ADM low and high expression groups. When the gene expression level of ADM (log2) was greater than 9.940702, these patients were classified into the ADM high expression group (n=114) and the rest were classified into the ADM low expression group (n=94). Then survival analysis was performed with Kaplan-Meier survival analysis. Log-rank test was used to determine the significance.

### Statistical analysis

Experimental data were presented as means ± standard deviation (SD). Statistical differences were assessed by a two-tailed Student's *t*-test; *p* < 0.05 was considered to be significant.

## SUPPLEMENTARY DATA, FIGURES AND TABLES




