# Supplementary material for: Adrenomedullin promotes the growth of pancreatic ductal adenocarcinoma through recruitment of myelomonocytic cells
Source: Oncotarget. 2016 Jul 4;7(34):55043–56. doi: 10.18632/oncotarget.10393 (PMC5342400; doi:10.18632/oncotarget.10393)
Supplement: Supplementary file 1 [file oncotarget-07-55043-s001.pdf]

Adrenomedullin promotes the growth of pancreatic ductal adenocarcinoma through recruitment of myelomonocytic cells

SUPPLEMENTARY DATA

SUPPLEMENTARY MATERIALS AND METHODS

Antibodies

Antibodies of CRLR, RAMP2, RAMP3, p38, p-p38, MMP-9 and MMP-2 were from Santa Cruz Biotechnology (Santa Cruz, CA). GPR182 antibody was from Bioworld (Nanjing, China). Antibodies for detecting eNOS, Erk1/2, p-Erk1/2, Akt, p-Akt, FAK, p-FAK, Src and p-Src were from Cell Signaling Technology (Beverley, MA). HIF-1α antibody was from GeneTex. CD31 and CD11b antibodies for immunofluorescence were from BD Bioscience (San Jose, CA). p-eNOS antibody and CD31 antibody for immunohistochemistry were from Abcam (Cambridge, UK). Antibodies for flow cytometry such as CD11b-FITC, CD11b-PE-Cy7, Integrin α5-PE, Integrin α2-FITC, VCAM-1-FITC and ICAM-1-FITC were from eBiosciences (San Diego, CA). Other antibodies for flow cytometry such as F4/80-FITC, CD206-APC were from Biolegend (San Diego, CA). FITC-linked anti-rat and anti-rabbit IgG antibodies were from Beijing Cowin Biotech (Beijing, China).

RNA extraction and mRNA analysis

Total RNA from cells was isolated with TRIzol (Invitrogen, Carlsbad, CA) and subjected to cDNA synthesis with the First Strand cDNA Synthesis Kit (Fermentas, Hanover, MD). qRT-PCR was performed with 2×TransStart Green qPCR SuperMix (TransGen Biotech, Beijing, China). The reaction was ran on the Mx3000P Real-Time PCR system (Stratagene, La Jolla, CA). GAPDH served as the loading control. Relative quantification was analyzed with the ΔΔCt method. All primers were listed in the Table S4.

Cell functional assays

For transwell migration assay, 1×10^5 myelomonocytic cells were seeded in the upper chamber of Millicell (Millipore, Bedford, MA) with DMEM containing 1% FBS. ADM or AMA and the same medium were added to the lower chamber. Cells were allowed to migrate for 6 h at 37°C and 5% CO_2. After fixed and stained with crystal violet (Sigma-Aldrich), migrated cells in five random fields were counted under the Olympus IX71 optical microscope.

For invasion assay, Millicell was pre-coated with GFR-Matrigel (Corning) for 30 min at 37°C. Then 1×10^5 myelomonocytic cells were seeded in the upper chamber and indicated reagents were added to the lower chamber. Cells were allowed to migrate for 24 h at 37°C and 5% CO_2. Migrated cells were fixed, stained with crystal violet and counted under the Olympus IX71 optical microscope.

For cell-cell adhesion assay, MS1 cells were plated in 24-well plate and allowed to form a confluent monolayer. Then the medium was replaced by DMEM without serum and cultured for 24 h. ADM or AMA was added to treat MS1 for 12 h. After washing MS1 with PBS twice, $CD11b^+$ myelomonocytic cells (1×10^5/well) from GFP-transgenic mice were added to co-culture with MS1 for 1 h. Unattached myelomonocytic cells were removed by washing with PBS thrice. Cells were fixed with 4% paraformaldehyde and five random fields were counted under the fluorescence microscope.

For cell-ECM adhesion assay, wells of 96-well plate were coated with Matrigel at 4°C overnight. Then these wells were blocked with 10 mg/mL BSA. After rinsing twice with RPMI 1640, myelomonocytic cells (1×10^5/well) were added to the wells and incubated for 1 h. Unattached cells were washed away by PBS thrice. Remaining cells were fixed with 5% glutaraldehyde and stained with 0.1% crystal violet. Solubilizing dye in acetic acid and the absorbance at 570 nm was measured using a plate reader (Thermo Fisher Scientific).

For trans-endothelial migration assay, MS1 cells were seeded in the Millicell and allowed to grow to confluence. MS1 was starved for 24 h and then treated with ADM for 12 h. After washing MS1 with PBS once, 1×10^5 GFP^+ myelomonocytic cells were added to migrate through MS1 for 6 h at 37°C and 5% CO_2. After fixed by 4% paraformaldehyde, trans-migrated myelomonocytic cells were counted in five random fields in each experiment under the fluorescence microscopy.

Cell proliferation assay

Pancreatic cancer cells were seeded in the wells of 96-well plate at a final density of 4×10^3 cells/well. Cells were treated with 1mg/mL PBS or clodronate liposomes for 24 h and 48 h. After the treatment, 10 μL MTT solution (Beyotime Biotechonology, China) was added to each well and the plate was incubated at 37°C for 4 h. Then 100 μL formazan dissolving solution was added to each well and incubated at 37°C for another 4 h. At last the absorbance at a wavelength of 570 nm (OD_{570}) was measured in the plate reader.

Cell apoptosis assay

Annexin V-FITC apoptosis detection kit (Beyotime Biotechnology) was used to detect cell apoptosis. Briefly, cells were harvested and washed with PBS once. Then cells were suspended in 300 μL binding buffer. 5 μL of Annexin-FITC was added to the cells and incubated for 15 min at room temperature in darkness, then 10 μL of propidium iodide (PI) was added to the cells and incubated for 5 min in darkness. Finally, 200 μL binding buffer was added and these cells were subjected to flow cytometry analysis.

Isolation and treatment of BMDM

Tibia and femur of C57BL/6 mice were flushed with 10 mL DMEM. After lysis of red blood cells, cells from bone marrow were incubated in DMEM supplemented with 10% FBS and 10 ng/mL M-CSF (Sino Biological, Beijing, China) for 1 week. BMDMs were treated with ADM or PANC-1 CM for 24 h.

Production of lentivirus and cell infection

The package plasmids of lentivirus (including PR 8.74, pVSVG, and pMCSSV or pSGEP) were co-transfected into HEK293T cells. 4 h before transfection, the medium of HEK293T cells was changed to DMEM without serum. Then plasmids were mixed with PEI reagent (Polysciences, USA) and added to HEK293T cells. 6 h later, DMEM was changed to the medium containing 20% FBS. The viral supernatant was collected 48 h after the transfection and filtered through a 0.45 μm filter. Then the supernatant was added to infect pancreatic cancer cells and stable cell lines were screened by adding blasticidin or puromycin (Invitrogen).

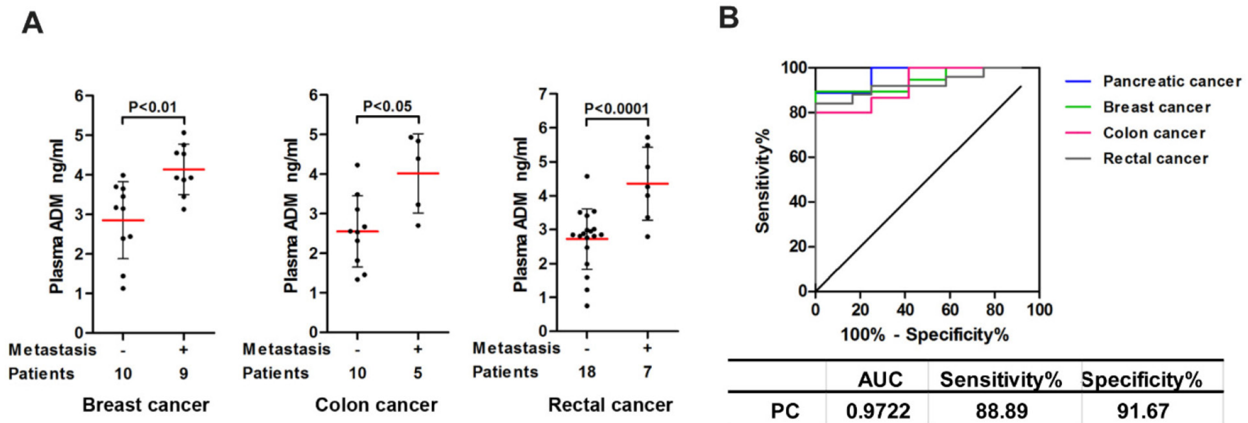

	AUC	Sensitivity%	Specificity%
PC	0.9722	88.89	91.67
BC	0.9474	89.47	91.67
CC	0.9278	80	91.67
RC	0.9300	84	91.67

Supplementary Figure S1: ADM correlates with tumor malignance. A. The plasma ADM levels correlated with tumor malignance in breast cancer and colorectal cancer. ADM in the plasma of non-metastatic and metastatic cancer patients was detected by ELISA. **B.** ROC curves of ADM in different clinical samples; the accuracy of diagnose in terms of sensitivity and specificity was presented. Data were representative of means ± SD.

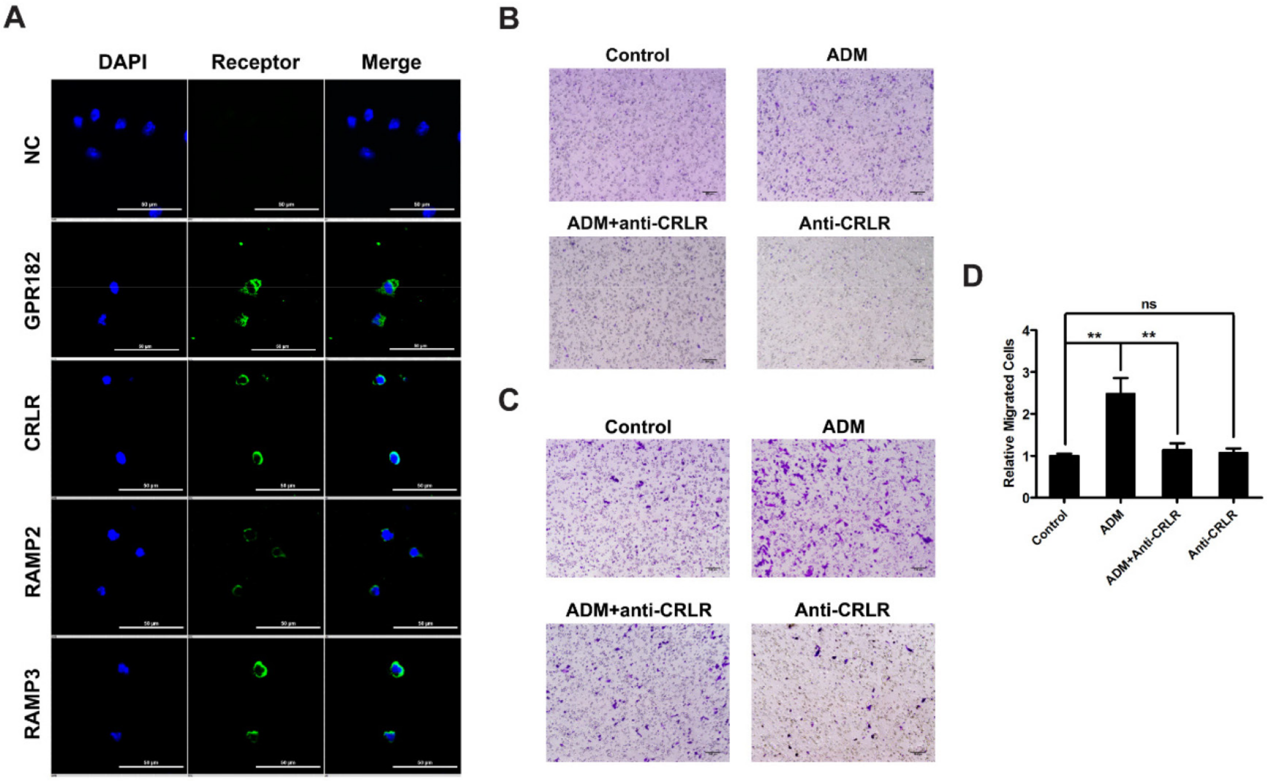

Supplementary Figure S2: ADM recruits myelomonocytic cells to pancreatic cancer by promoting the migration and invasion of myelomonocytic cells. A. Immunofluorescence showing the expression of ADM receptor components in $CD11b^+$ myelomonocytic cells; Scale bar=50µm. **B.** Representative images of the effect of CRLR blockade with its antibody on ADM-induced myelomonocytic cell migration; Scale bar=100µm. **C.** Representative images of the effect of CRLR blockade with its antibody on ADM-induced Raw 264.7 cell migration; Scale bar=100µm. **D.** Quantified result of (C). Data were representative of means ± SD from at least three independent experiments. *p* value: Student's *t*-test; $^{**}p < 0.01$, ns: not significant.

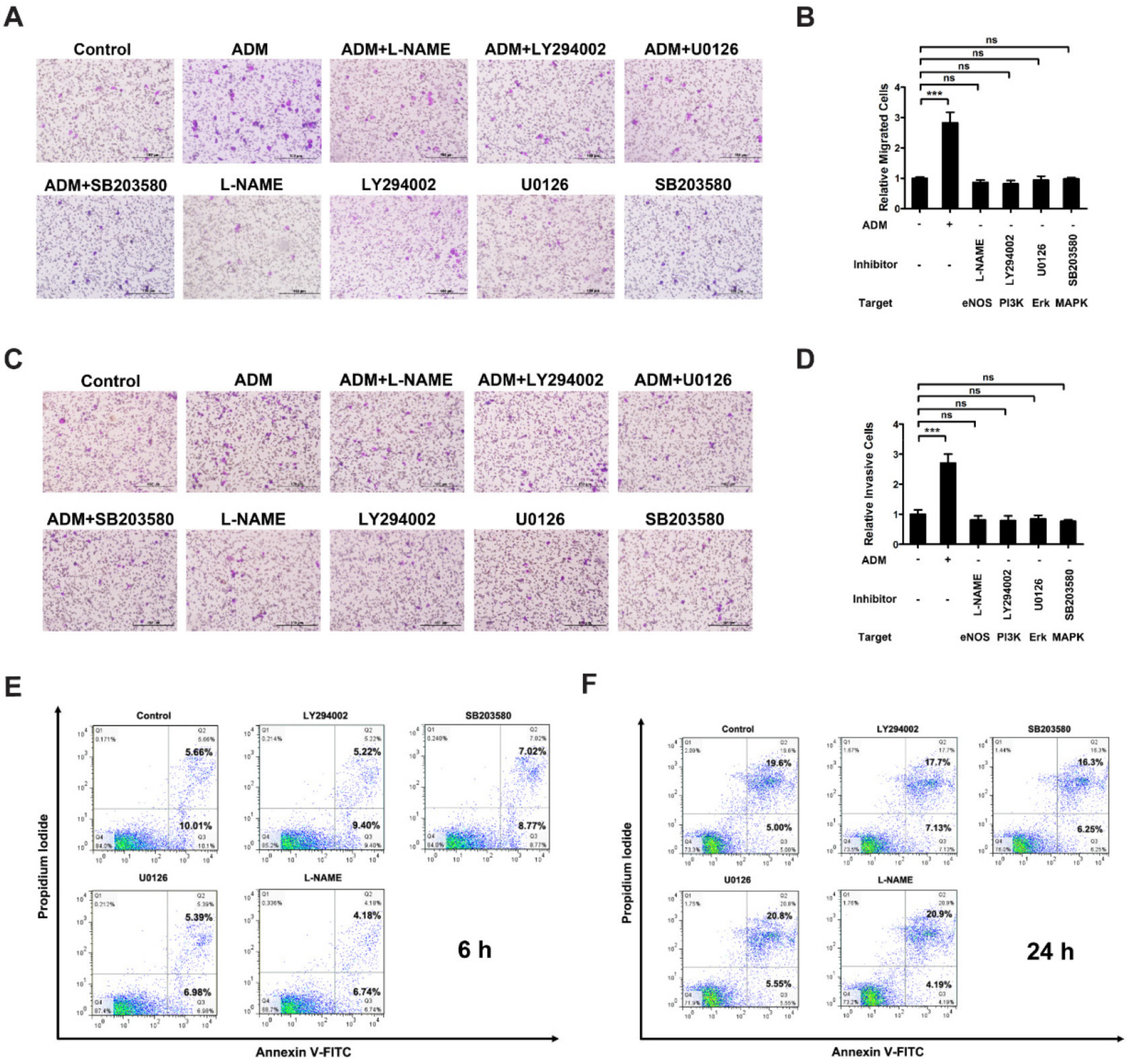

Supplementary Figure S3: Inhibitors of PI3K, MAPK and eNOS can inhibit ADM-induced migration and invasion of myelomonocytic cells. A. Representative images of the effects of L-NAME, LY294002, U0126 and SB203580 on ADM-induced myelomonocytic cell migration; Scale bar=100 μm. **B.** Quantitation result showing the migration ability of myelomonocytic cells upon pharmacological inhibition of eNOS, PI3K, Erk1/2, and p38 in the absence of ADM. **C.** Representative images of the effects of L-NAME, LY294002, U0126 and SB203580 on ADM-induced myelomonocytic cell invasion; Scar bar=100μm. **D.** Quantitation result showing the invasion ability of myelomonocytic cells upon pharmacological inhibition of eNOS, PI3K, Erk1/2, and p38 in the absence of ADM. **E.** Flow cytometry data showing the apoptosis of myelomonocytic cells upon pharmacological inhibition of eNOS, PI3K, Erk1/2, and p38 for 6 h. **F.** Flow cytometry data showing the apoptosis of myelomonocytic cells upon pharmacological inhibition of eNOS, PI3K, Erk1/2, and p38 for 24 h. Data were representative of means ± SD from at least three independent experiments. *p* value: Student's *t*-test; $^{***}p < 0.001$, ns: not significant.

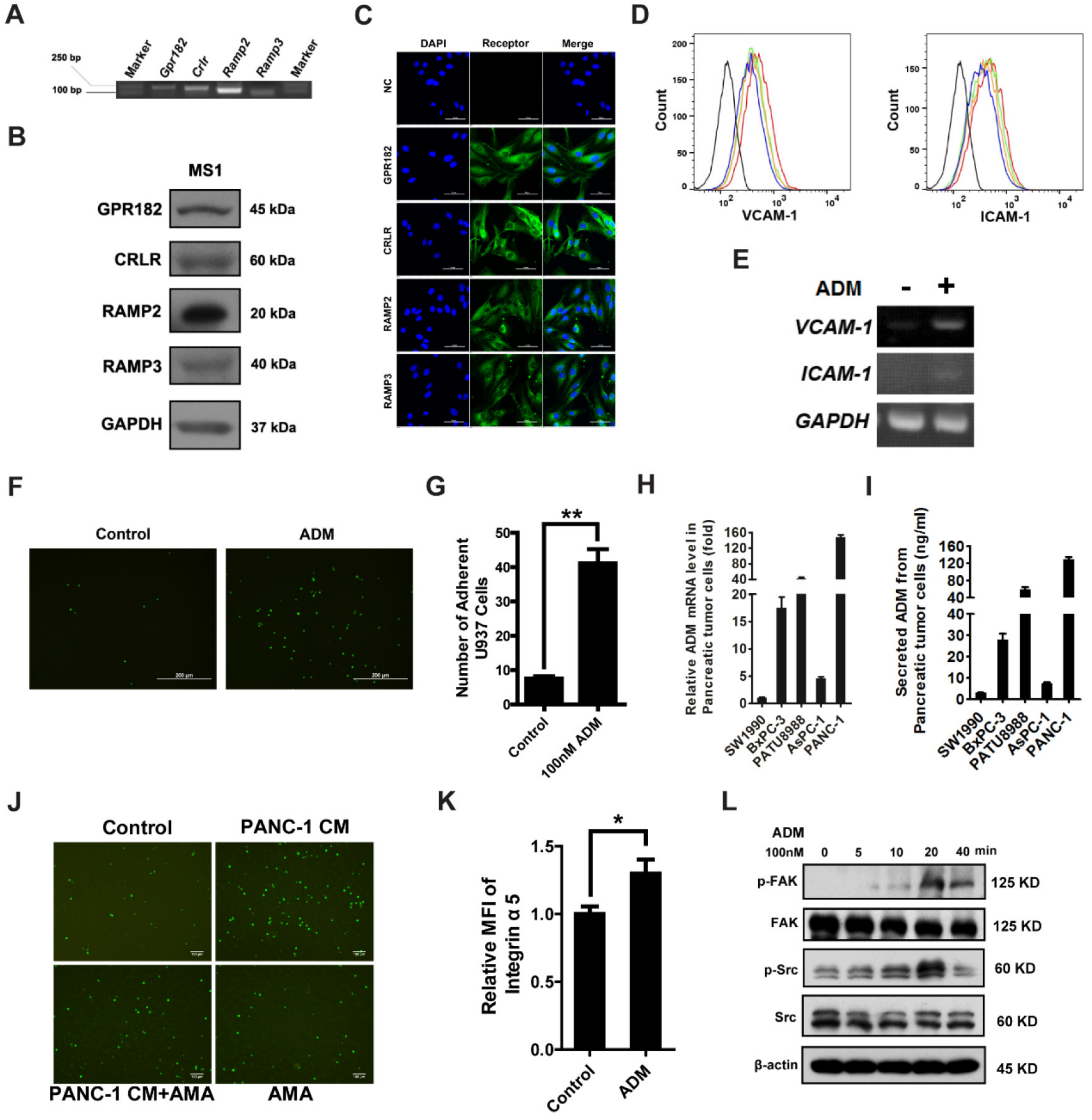

Supplementary Figure S4: ADM enhances the adhesion of myelomonocytic cells to endothelial cells and extracellular matrix (ECM). A. RT-PCR showing the expression of ADM receptor components in MS1. **B.** Western blot showing the expression of ADM receptor components in MS1. **C.** Immunofluorescence showing the expression of ADM receptor components in MS1; Scale bar=50 µm. **D.** Flow cytometry analysis showing the effect of ADM on the expression of VCAM-1 and ICAM-1 in MS1. **E.** RT-PCR displaying the effect of ADM on the expression of VCAM-1 and ICAM-1 in HUVECs. **F.** Representative images of ADM promoting the adhesion of U937 cells to HUVECs. U937 cells labeled with CellTracker Green were added to ADM-treated HUVECs and incubated for 1h. Unattached cells were washed away with PBS for 3 times. Five independent fields were observed under the fluorescence microscope; Scale bar=100 µm. **G.** Quantified results of (F). **H.** mRNA levels of ADM in five different pancreatic cancer cell lines. **I.** Quantitation of ADM in the conditioned medium of five different pancreatic cancer cell lines by ELISA. **J.** Representative images of PANC-1 CM-induced myelomonocytic cell adhesion to MS1 either alone or in the presence of AMA. **K.** The effect of ADM on the expression of integrin α5 in myelomonocytic cells. **L.** Western blot showing the effect of ADM on inducing the phosphorylation of FAK and Src in myelomonocytic cells upon treatment of 100 nM ADM with indicated time. Data were representative of means ± SD from at least three independent experiments. *p* value: Student's *t*-test; $^{*}p < 0.05$, $^{**}p < 0.01$.

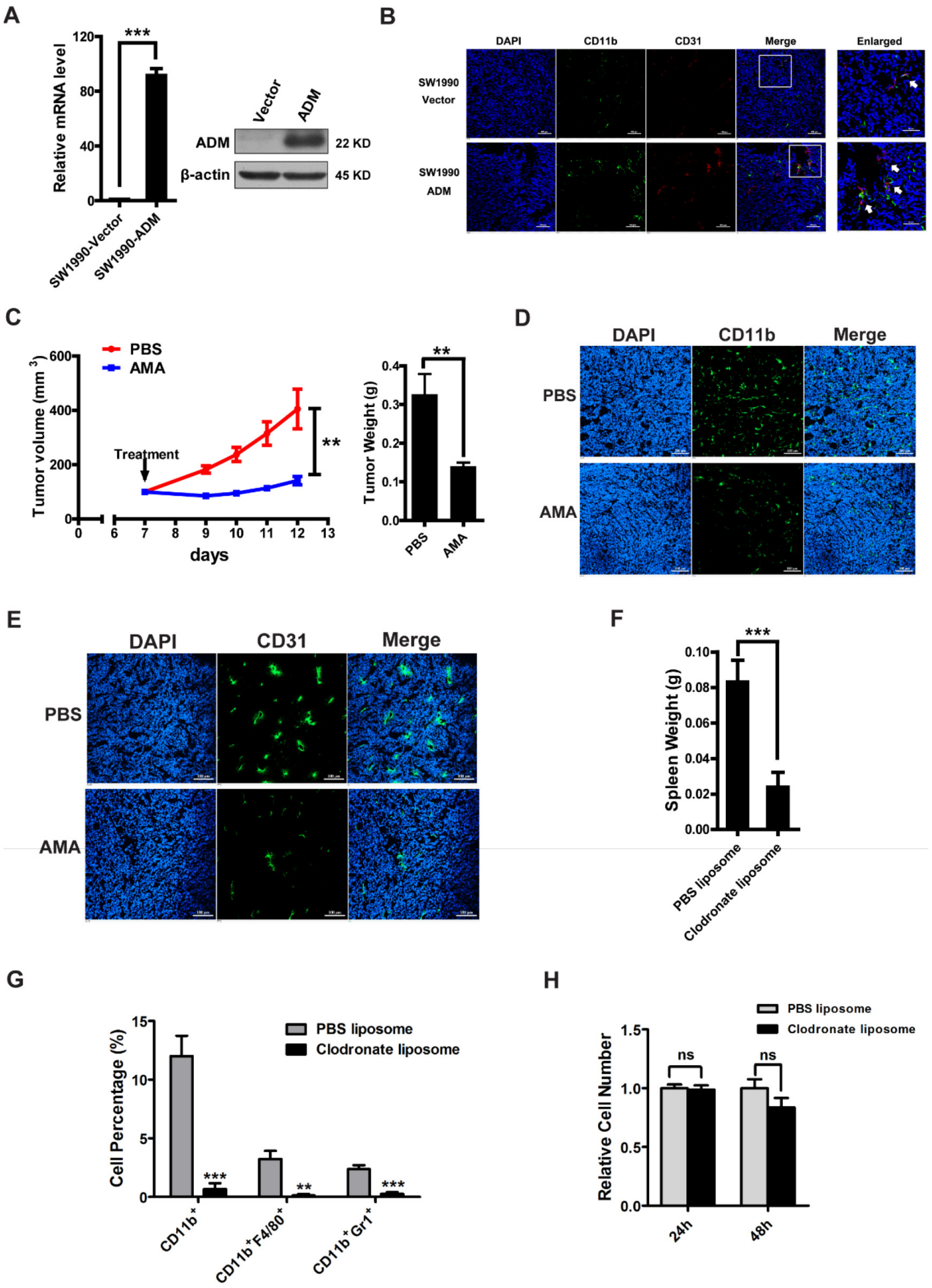

Supplementary Figure S5: ADM promotes the recruitment of CD11b$^+$ myelomonocytic cells and tumor angiogenesis in SW1990-ADM tumor-bearing mice. A. The mRNA (left) and protein (right) levels of ADM in SW1990-Vector and SW1990-ADM cells. **B.** Adhesion of CD11b$^+$ myelomonocytic cells to CD31$^+$ endothelial cells in SW1990-Vector and SW1990-ADM tumor tissues; Scale bar=100 μm and 50 μm for enlarged field. **C.** SW1990-ADM tumor growth (left) and tumor weight (right) in mice treated with PBS or AMA (n=6 mice/group). **D.** Representative immunofluorescence images of the effect of AMA on the recruitment of CD11b$^+$ myelomonocytic cells in SW1990-ADM tumor-bearing mice; Blue: DAPI, green: CD11b; Scale bar=100 μm. **E.** Representative immunofluorescence images of the effect of AMA on the tumor angiogenesis in SW1990-ADM tumor-bearing mice; Blue: DAPI, green: CD31; Scale bar=100 μm. **F.** The weight of mice spleens after treatment with PBS or clodronate liposomes. **G.** The percentage of myelomonocytic cells in the spleens of SW1990-ADM tumor-bearing mice after treatment with PBS or clodronate liposomes. After the treatment with PBS or clodronate liposomes, the whole suspension of splenocytes were stained with CD11b-PECy/7, F4/80-FITC and Gr-1-PE antibodies. Then the cell percentage was detected by flow cytometry. **H.** The effect of PBS or clodronate liposomes on cell viability of SW1990-ADM cells. Data were representative of means ± SD or SEM for animal experiment. *p* value: Student's *t*-test; $^{**}p < 0.01$, $^{***}p < 0.001$, ns: not significant.

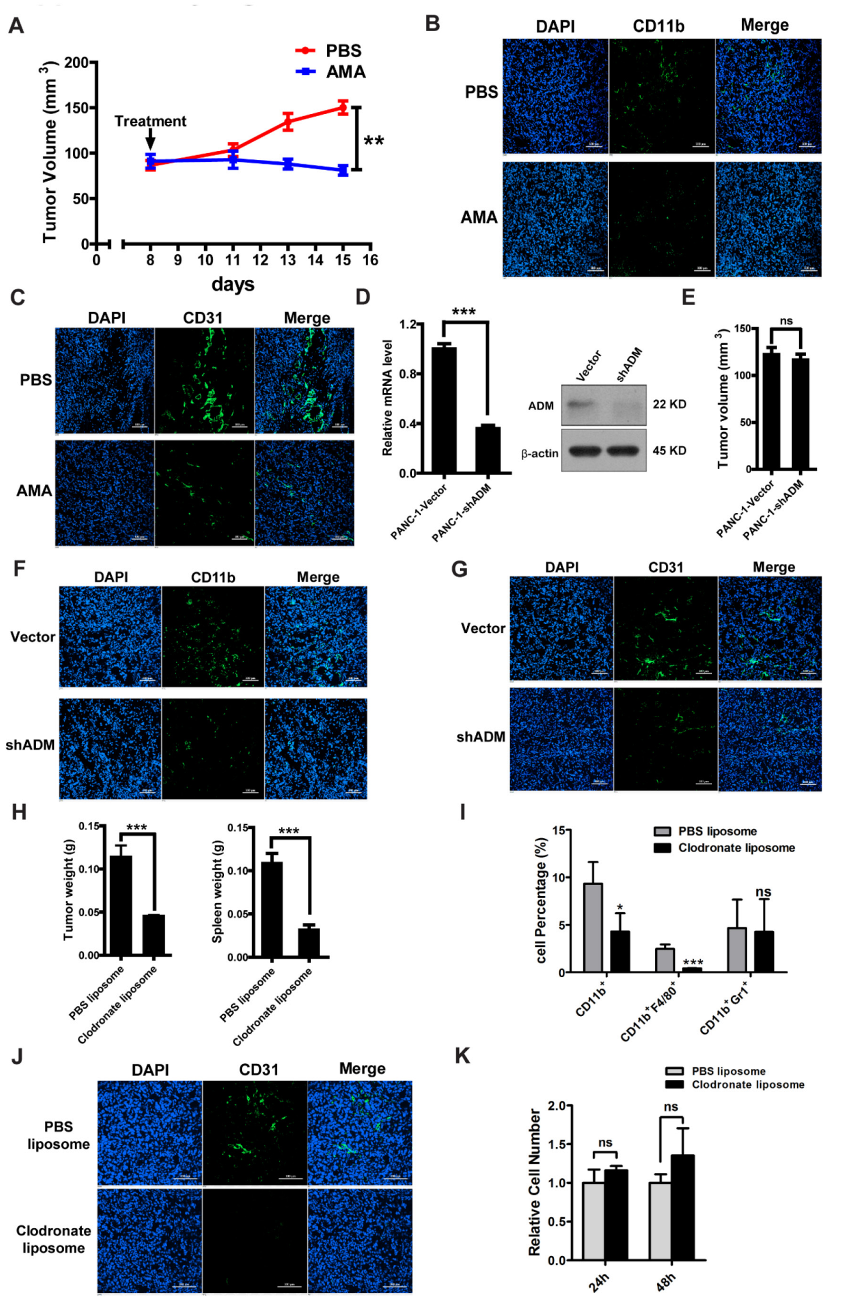

Supplementary Figure S6: Knock down of ADM inhibits the recruitment of CD11b$^+$ myelomonocytic cells and tumor angiogenesis in PANC-1 tumor-bearing mice. A. PANC-1 tumor growth in mice treated with PBS or AMA (n=5 mice/group). **B.** Representative immunofluorescence images of the effect of AMA on the recruitment of CD11b$^+$ myelomonocytic cells in PANC-1 tumor-bearing mice; Blue: DAPI, green: CD11b; Scale bar=100 μm. **C.** Representative immunofluorescence images of the effect of AMA on the tumor angiogenesis in PANC-1 tumor-bearing mice; Blue: DAPI, green: CD31; Scale bar=100 μm. **D.** The mRNA (left) and protein (right) levels of ADM in PANC-1-Vector and PANC-1-shADM cells. **E.** PANC-1-Vector and PANC-1-shADM tumor volumes in mice after 1 week. **F.** Representative immunofluorescence images of the effect of ADM on the recruitment of CD11b$^+$ myelomonocytic cells in PANC-1-Vector and PANC-1-shADM tumor-bearing mice; Blue: DAPI, green: CD11b; Scale bar=100 μm. **G.** Representative immunofluorescence images of the effect of ADM on tumor angiogenesis in PANC-1-Vector and PANC-1-shADM tumor-bearing mice; Blue: DAPI, green: CD31; Scale bar=100 μm. **H.** PANC-1 tumor weight (left) and spleen weight (right) in mice treated with PBS or clodronate liposomes. **I.** The percentage of myelomonocytic cells in the spleens of PANC-1 tumor-bearing mice after treatment with PBS or clodronate liposomes. **J.** Representative immunofluorescence images of the effect of clodronate liposomes on the tumor angiogenesis in PANC-1 tumor-bearing mice; Blue: DAPI, green: CD31; Scale bar=100 μm. **K.** MTT assay showing the effect of PBS or clodronate liposomes on cell viability of PANC-1 cells. Data were representative of means ± SD or SEM for animal experiment. *p* value: Student's *t*-test; $^{*}p < 0.05$, $^{**}p < 0.01$, $^{***}p < 0.001$, ns: not significant.

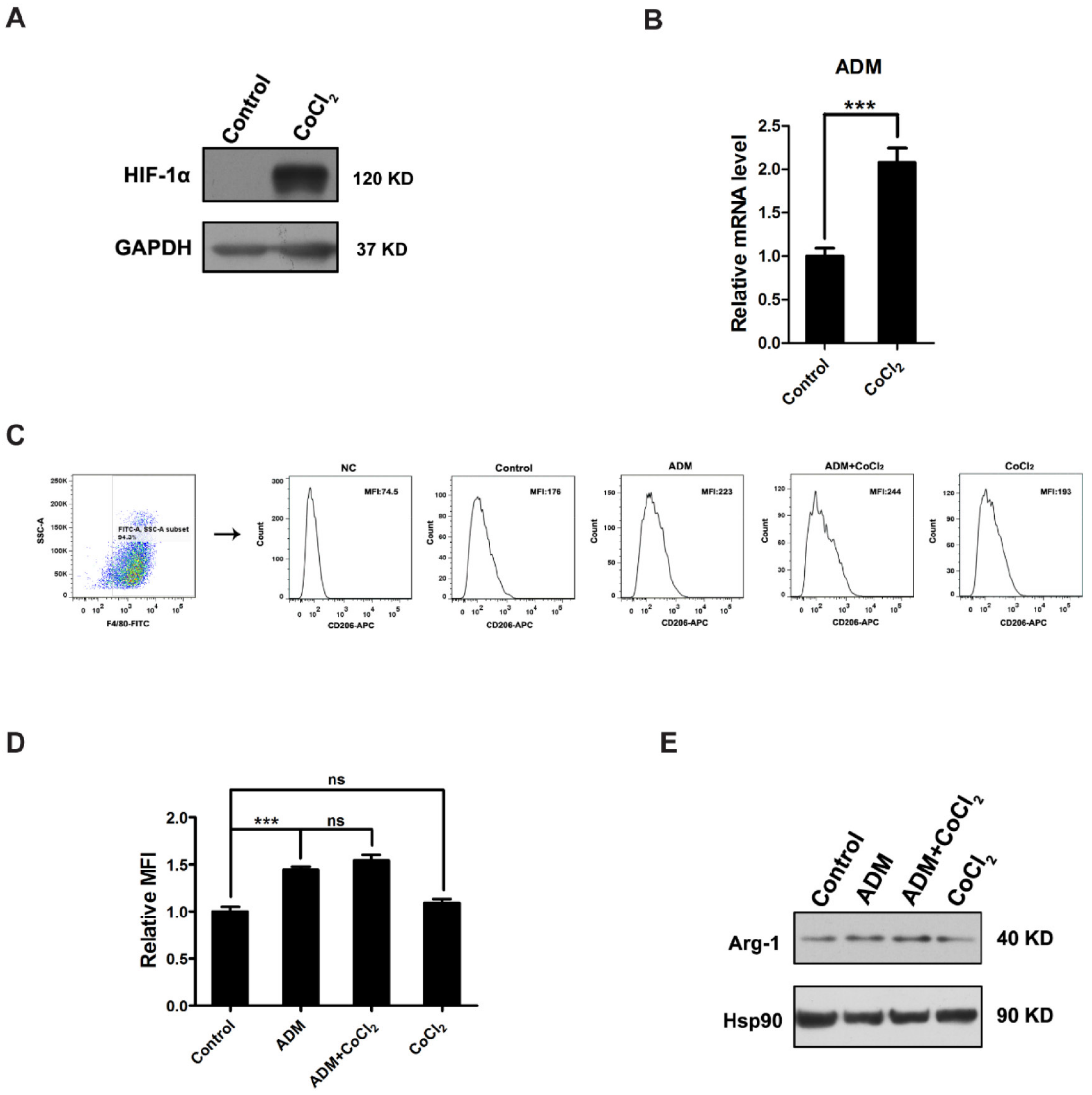

Supplementary Figure S7: The effect of hypoxia-mimetic agent $CoCl_2$ on the process of ADM educating BMDMs towards pro-tumor types. A. Western blot showing the effect of 100 µM $CoCl_2$ treatment on expression of HIF-1α in BMDMs. **B.** qRT-PCR showing the effect of $CoCl_2$ treatment on expression of ADM in BMDMs. **C.** Flow cytometry analysis showing the effect of $CoCl_2$ treatment on the process of ADM stimulating BMDMs towards M2 type. **D.** Quantified result of MFI in (C). **E.** Western blot showing the effect of $CoCl_2$ treatment on the process of ADM stimulating BMDMs towards M2 type. Data were representative of means ± SD from at least three independent experiments. *p* value: Student's *t*-test; $^{***}p < 0.001$, ns: not significant.

Supplementary Table S1: The correlation between mRNA levels of ADM and clinicopathological status of pancreatic cancer patients

		Total samples	ADM Low	ADM High	*p* value
**Age (year)**	**≥ 67**	**130**	**60**	**70**	**> 0.05**
	**< 67**	**106**	**49**	**57**	
**Gender**	**male**	**125**	**54**	**71**	**> 0.05**
	**female**	**111**	**55**	**56**	
**Lymph node metastasis stage**	**N0**	**55**	**29**	**26**	**> 0.05**
	**N1**	**175**	**77**	**98**	

The mRNA levels of ADM from ICGC were clustered into different groups. Low and high mRNA levels of ADM in different ages and genders of pancreatic cancer patients or different stages of lymph node metastasis were analyzed. Chi-square test was used to assess the statistical significance.

Supplementary Table S2: The correlation between mRNA levels of ADM and pancreatic cancer differentiation

	Total samples	Well differentiated	Moderately differentiated	Poorly differentiated	Undifferentiated	*p* value
ADM Low	108	12	65	28	3	< 0.01
ADM High	123	2	73	47	1	

The mRNA levels of ADM from ICGC were clustered into groups. The statistical significance of correlation between ADM expression levels and pancreatic cancer differentiation was assessed by Chi-square test.

Supplementary Table S3: Tumor composition of 68 human plasma samples

See Supplementary File 1

Supplementary Table S4: Oligonucleotide primer sequences used for RT-PCR

Genes	Species	Oligonucleotide	Sequence
Crlr	Mouse	Upper primer<br>Lower primer	5' –TGGCTTTTCCCACTCTGAT– 3'<br>5' –TCACATCACTAGATCATACAT– 3'
Ramp2	Mouse	Upper primer<br>Lower primer	5' –CAGAATCAATCTCATCCCACTGAG – 3'<br>5' –GTCCATGCAACTCTTGTACTCATACC – 3'
Ramp3	Mouse	Upper primer<br>Lower primer	5' –TGCAACGAGACAGGGATGC – 3'<br>5' –GCATCATGTCAGCGAAGGC – 3'
Gpr182	Mouse	Upper primer<br>Lower primer	5' - CCGTTACCTTCCCAAGGA - 3'<br>5' - TTAGCTGGCTACAGAATTGCA - 3
Vcam-1	Mouse	Upper primer<br>Lower primer	5' –GCTGCGAGTCACCATTGTTCTC – 3'<br>5' –ACCACCCTCTTGAAGCCTTGTG – 3'
Icam-1	Mouse	Upper primer<br>Lower primer	5' –CCTGTTTCCTGCCTCTGAAG – 3'<br>5' –GTCTGCTGAGACCCCTCTTG – 3'
Sele	Mouse	Upper primer<br>Lower primer	5' –GTGCGGTGTACGTCCTCTGG – 3'<br>5' –GACTTGTAGGTGAATTCTCCAGTAGT – 3'
VCAM-1	Human	Upper primer<br>Lower primer	5' –GGATTCTGTGCCCACAGTA– 3'<br>5' –CCTGGCTCAAGCATGTCATA– 3'
ICAM-1	Human	Upper primer<br>Lower primer	5' –GGCAAGAACCTTACCCTA– 3'<br>5' –CATTCAGCGTCACCTTGG– 3'
SELE	Human	Upper primer<br>Lower primer	5' –CTCTGACAGAAGAAGCCAAGAAC– 3'<br>5' –ACTTGAGTCCACTGAAGCCAGG – 3'
GAPDH	Human & Mouse	Upper primer<br>Lower primer	5' –CAAGGTCATCCATGACAACTTTG– 3'<br>5' –GTCCACCACCCTGTTGCTGTAG– 3'

Oligonucleotide primer sequences used for qRT-PCR

Genes	Species	Oligonucleotide	Sequence
ADM	Human	Upper primer<br>Lower primer	5' –CCTGATGTACCTGGGTTCG– 3'<br>5' –TTCCACTTCTTTCGAAACTCC– 3'
Adm	Mouse	Upper primer<br>Lower primer	5' –CACCCTGATGTTATTGGGTTCA– 3'<br>5' –CCACTTATTCCACTTCTTTCGGA– 3'
Arg-1	Mouse	Upper primer<br>Lower primer	5' –CTCCAAGCCAAAGTCCTTAGAG– 3'<br>5' –AGGAGCTGTCATTAGGGACATC– 3'
iNOS	Mouse	Upper primer<br>Lower primer	5' –CCACCTCTATCAGGAAGAAA– 3'<br>5' –CTGCACCGAAGATATCTTCA– 3'
Vcam-1	Mouse	Upper primer<br>Lower primer	5' –GCCCACTAAACGCGAAGGT– 3'<br>5' –ACTGGGTAAATGTCTGGAGCC– 3'
Icam-1	Mouse	Upper primer<br>Lower primer	5' –TGCCTCTGAAGCTCGGATATAC– 3'<br>5' –TCTGTCGAACTCCTCAGTCAC– 3'
GAPDH	Human & Mouse	Upper primer<br>Lower primer	5' –CAAGGTCATCCATGACAACTTTG– 3'<br>5' –GTCCACCACCCTGTTGCTGTAG– 3'
