# Supplementary material for: Adrenomedullin promotes the growth of pancreatic ductal adenocarcinoma through recruitment of myelomonocytic cells
Source: Oncotarget. 2016 Jul 4;7(34):55043–56. doi: 10.18632/oncotarget.10393 (PMC5342400; doi:10.18632/oncotarget.10393)
Supplement: Supplementary file 2 [file oncotarget-07-55043-s002.doc]

**Supplementary Table 4**

| **No.** | **Gender** | **Age** | **Organ** | **Metastasis** |
| --- | --- | --- | --- | --- |
| 1 | **F** | **76** | **pancreas** | **-** |
| 2 | **F** | **47** | **pancreas** | **-** |
| 3 | **M** | **54** | **pancreas** | **-** |
| 4 | **M** | **40** | **pancreas** | **-** |
| 5 | **F** | **70** | **pancreas** | **-** |
| 6 | **F** | **65** | **pancreas** | **-** |
| 7 | **M** | **54** | **pancreas** | **-** |
| 8 | **M** | **53** | **pancreas** | **-** |
| 9 | **M** | **44** | **pancreas** | **-** |
| 10 | **F** | **52** | **breast** | **-** |
| 11 | **F** | **74** | **breast** | **-** |
| 12 | **F** | **50** | **breast** | **-** |
| 13 | **F** | **49** | **breast** | **-** |
| 14 | **F** | **52** | **breast** | **-** |
| 15 | **F** | **61** | **breast** | **-** |
| 16 | **F** | **65** | **breast** | **-** |
| 17 | **F** | **55** | **breast** | **-** |
| 18 | **F** | **48** | **breast** | **-** |
| 19 | **F** | **56** | **breast** | **-** |
| 20 | **F** | **77** | **breast** | **+** |
| 21 | **F** | **56** | **breast** | **+** |
| 22 | **F** | **52** | **breast** | **+** |
| 23 | **F** | **61** | **breast** | **+** |
| 24 | **F** | **52** | **breast** | **+** |
| 25 | **F** | **42** | **breast** | **+** |
| 26 | **F** | **51** | **breast** | **+** |
| 27 | **F** | **71** | **breast** | **+** |
| 28 | **F** | **63** | **breast** | **+** |
| 29 | **M** | **73** | **colon** | **-** |
| 30 | **M** | **54** | **colon** | **-** |
| 31 | **F** | **75** | **colon** | **-** |
| 32 | **M** | **92** | **colon** | **-** |
| 33 | **F** | **30** | **colon** | **-** |
| 34 | **M** | **66** | **colon** | **-** |
| 35 | **M** | **73** | **colon** | **-** |
| 36 | **M** | **63** | **colon** | **-** |
| 37 | **M** | **28** | **colon** | **-** |
| 38 | **M** | **77** | **colon** | **-** |
| 39 | **F** | **49** | **colon** | **+** |
| 40 | **F** | **76** | **colon** | **+** |
| 41 | **F** | **59** | **colon** | **+** |
| 42 | **F** | **47** | **colon** | **+** |
| 43 | **M** | **72** | **colon** | **+** |
| 44 | **F** | **62** | **rectal** | **-** |
| 45 | **M** | **57** | **rectal** | **+** |
| 46 | **M** | **85** | **rectal** | **-** |
| 47 | **F** | **80** | **rectal** | **-** |
| 48 | **F** | **63** | **rectal** | **-** |
| 49 | **M** | **68** | **rectal** | **-** |
| 50 | **M** | **36** | **rectal** | **-** |
| 51 | **F** | **51** | **rectal** | **+** |
| 52 | **M** | **53** | **rectal** | **+** |
| 53 | **M** | **53** | **rectal** | **-** |
| 54 | **F** | **51** | **rectal** | **-** |
| 55 | **F** | **71** | **rectal** | **-** |
| 56 | **F** | **34** | **rectal** | **+** |
| 57 | **M** | **45** | **rectal** | **-** |
| 58 | **F** | **70** | **rectal** | **-** |
| 59 | **M** | **51** | **rectal** | **+** |
| 60 | **M** | **46** | **rectal** | **-** |
| 61 | **M** | **45** | **rectal** | **-** |
| 62 | **F** | **62** | **rectal** | **+** |
| 63 | **M** | **43** | **rectal** | **-** |
| 64 | **F** | **67** | **rectal** | **-** |
| 65 | **M** | **44** | **rectal** | **-** |
| 66 | **M** | **47** | **rectal** | **+** |
| 67 | **M** | **61** | **rectal** | **-** |
| 68 | **M** | **78** | **rectal** | **-** |

**Table S4: Tumor composition of 68 human plasma samples.**
